# A cluster randomised controlled trial of nurse and GP partnership for care of Chronic Obstructive Pulmonary Disease

**DOI:** 10.1186/1471-2466-8-8

**Published:** 2008-06-03

**Authors:** Nicholas Zwar, Oshana Hermiz, Iqbal Hasan, Elizabeth Comino, Sandy Middleton, Sanjyot Vagholkar, Guy Marks

**Affiliations:** 1 Centre for Primary Health Care and Equity, University of New South Wales, Sydney, Australia; 2 School of Nursing – NSW/ACT, Australian Catholic University, Sydney, Australia; 3 General Practice Unit, Sydney South West Area Health Service, Sydney, Australia; 4 Chest Clinic, Liverpool Health Service, Sydney, Australia

## Abstract

**Background:**

Chronic obstructive pulmonary disease (COPD) is a significant health problem worldwide. This randomised controlled trial aims at testing a new approach that involves a registered nurse working in partnership with patients, general practitioners (GPs) and other health professionals to provide care to patients according to the evidence-based clinical practice guidelines. The aim is to determine the impact of this partnership on the quality of care and patient outcomes.

**Methods:**

A cluster randomised control trial design was chosen for this study. Randomisation occurred at practice level. GPs practising in South Western Sydney, Australia and their COPD patients were recruited for the study.

The intervention was implemented by nurses specifically recruited and trained for this study. Nurses, working in partnership with GPs, developed care plans for patients based on the Australian COPDX guidelines. The aim was to optimise patient management, improve function, prevent deterioration and enhance patient knowledge and skills. Control group patients received 'usual' care from their GPs.

Data collection includes patient demographic profiles and their co-morbidities. Spirometry is being performed to assess patients' COPD status and CO analyser to validate their smoking status. Patients' quality of life and overall health status are being measured by St George's Respiratory Questionnaire and SF-12 respectively. Other patient measures being recorded include health service use, immunisation status, and knowledge of COPD. Qualitative methods will be used to explore participants' satisfaction with the intervention and their opinion about the value of the partnership.

**Analysis:**

Analysis will be by intention to treat. Intra-cluster (practice) correlation coefficients will be determined and published for all primary outcome variables to assist future research. The effect of the intervention on outcomes measured on a continuous scale will be estimated and tested using mixed model analysis of variance in which time and treatment group will be fixed effects and GP practice and subject nested within practice will be random effects. The effect of the intervention on the dichotomous variables (such as smoking status, patient knowledge) will be analysed using generalised estimating equations with a logistic link and a model structure that is analogous to that described above.

**Trial registration:**

ACTRN012606000304538

## Background

### Significance of COPD

Chronic obstructive pulmonary disease (COPD) is a leading cause of disability, hospital admission and premature mortality in men and women [1]. According to WHO estimates, there are 210 million people worldwide who have COPD [2]. In 2005, there were 3 million deaths from COPD globally and the projection is that the mortality from COPD will increase by 30% over the next 10 years unless urgent actions are taken to combat it [2].

The prevalence of COPD (GOLD stage 2 or higher, that is post-bronchodilator FEV_1_/FVC ratio < 0.7 and FEV_1 _< 80% predicted) in a representative sample of people aged 40 years and over living in south-east Sydney in 2006 was 10.8%. This was in the mid-range of estimates for centres in 12 countries that participated in the first round of the BOLD international comparative study [3]. In recent years there has been a decline in death rates from COPD, but it still remains the fifth leading cause of death for males (40.6 deaths/100,000 population) and the seventh leading cause of death for women (19.5 deaths/100,00 population) in Australia [4].

In addition to the significant social and economic burden of deaths caused by COPD, the condition also poses a significant burden on the Australian health care system through hospitalisation of COPD patients with complications. In 2001–02, COPD led to 51,621 admissions Australia-wide, with an average stay of 7.5 days [3]. Nationwide, costs of COPD including hospitalisation, together with related community medical care, pharmaceutical services and indirect costs such as lost productivity are estimated to be in the range of 820 – 900 million Australian dollars annually [5].

### Evidence-based management

Guidelines for diagnosis and management of COPD are now available. In Australia the COPDX guidelines are commonly used which detail recommendations for preventing or slowing disease progression and optimising function for COPD patients [6]. The key recommendations include:

• Smoking cessation

• Pulmonary rehabilitation

• Influenza vaccination

• Optimising the use of inhaled bronchodilators & corticosteroids

• Patient education.

Despite the publication of the COPDX guidelines there is a low level of awareness about the existence of such guidelines among GPs in Australia [7]. To reduce the impact of COPD in Australia initiatives to encourage GP uptake of guideline recommendation are warranted.

### Role of General Practice in COPD management

General practice is well placed to provide early intervention and management of COPD patients. In Australia, general practice is the most common point of contact in the health system as 87% of people visit their GPs at least once a year [8]. COPD accounts for 0.6% of all problems managed in general practice [9]. While improved care in the community has the potential to substantially improve outcomes, research to date suggests that GPs experience difficulties providing care for patients with chronic illness under the current model of care which is largely encounter based and does not involve non-GP health providers [10]. There is a need for more structured systems to implement chronic illness care such as a multidisciplinary care plan. The use of care plans has become a recommendation for the management of people with chronic illnesses such as diabetes and asthma [11-13]. Similar to these patients, COPD patients often have complex needs and the COPDX guidelines[6] recommend the use of multidisciplinary care plans. However, research suggests that GPs need more external support to develop and implement multidisciplinary care plans for these patients [14-16].

### Role of nurses in care of COPD

A review of the roles of specialised nurses in care of patients with diabetes or COPD found evidence that they improved patient self care, quality of life and satisfaction [17]. A Cochrane review revealed that a nurse outreach program involving nurse home visits to COPD patients providing support and education, monitoring health status and providing liaison with physicians resulted in improved quality of life and reduced mortality [18]. A New Zealand study, that involved practice nurses and GPs implementing a care plan with advice from a respiratory nurse and specialist physician, resulted in reduced hospital admissions and reduced hospital bed days and there were significant improvements in spirometry [19].

### Lesson learnt from a previous study

A randomised controlled trial (RCT) [20] conducted in 1998–99 which evaluated the effect of a brief, nurse-led intervention at 1 week and 4 weeks interval after discharge from hospital on clinical outcomes in patients with COPD. The aim was to evaluate the efficacy of limited community based care provided by nurses to COPD patients. Results from this study demonstrated limited success. There were some improvements in activity and knowledge scores of patients in the intervention group and they were more satisfied with their care, but there was no impact on rates of emergency department presentation and hospital readmission. We subsequently attributed the failure of the intervention to three key reasons 1) GPs were not sufficiently involved in the patient care (evident by the fact that only 31% of the GPs recalled having received patient related information (care plan) from the nurses), 2) there was no formal partnership between GPs and nurses streamlining patient care, and 3) the unavailability of universally accepted guidelines for management of COPD such as, COPDX.

### Aim of the study

The aim of this study is to test a new approach to improving the care of patients with COPD managed in general practice. The intervention involves a registered nurse with specific training working in partnership with the patient, GPs and other health professionals to provide evidence-based care according to the Australian COPDX guidelines. The research aims to determine the impact of this partnership on the quality of care and health outcomes for patients with COPD at six and 12 months follow-up.

These are the hypotheses being tested:

1. That in patients with COPD, the intervention improves disease-related quality of life and overall health including patient health status, lung function and health service use.

2. That the intervention improves the quality of care provided to patients with COPD and that this will impact on knowledge, immunisation compliance, smoking cessation and satisfaction with care.

### Research Plan

A cluster randomised control trial design has been chosen for this study. As GPs find it difficult to selectively offer interventions to their patients, the cluster design was implemented to avoid contamination between intervention and control groups. Randomisation occurred at practice level and an adjustment to the sample size estimates allowed for clustering.

### Recruitment

#### General Practitioner/practice recruitment

GPs were recruited from a list of 256 GPs practising in South Western Sydney (SWS). The list includes GPs who have previously participated in research and/or attended local continuing medical education (CME) activities and also includes the chief investigators' personal contacts. Letters of invitation were sent to these GPs with a copy of the GP information statement. Within one to two weeks of the initial invitation telephone contacts were made with the GPs by one of the chief investigators to explain the study, check their eligibility, answer their questions about the study and formally invite them to participate. The GPs expressing interest were visited by the chief investigator at their practices. At the practice visit the details of involvement were explained and written consent was obtained. Inclusion criteria for GPs were: practising in SWS, use of an electronic prescribing system, and having seen COPD patients in the past 12 months. The aim was to recruit at least 40 GPs.

#### Patient recruitment

Participating GPs were asked to search their electronic prescription records and identify patients who had been prescribed medications for COPD. These were defined as inhaled beta 2-agonists, inhaled corticosteroids, ipratropium bromide, tiotropium, oral theophylline and oral corticosteroids. Patients' were eligible if they were aged between 40 and 80 years, prescribed one or more of the above medications, have seen the GP in the previous 12 months, and had a clinical diagnosis of either COPD, emphysema or chronic bronchitis. Patients who lived outside SWS, did not speak English, or had significant cognitive impairment were excluded from the study.

Letters were sent to the eligible patients inviting them to take part in the study. The letters included a patient information sheet and a consent form. Those who consented to take part returned the form to the research team. The project officer from the research team then contacted these patients and organised a home visit (or at the GP surgery if preferred by patient) to collect the baseline data as detailed below.

Two two-weekly reminders were sent to the non-responding patients. The aim was to recruit 400 patients from 40 GPs (an average 10 patients per GP).

### Randomisation

Practices were allocated to intervention and control groups according to a computer generated list comprising 11 randomly permuted blocks of size four. A system of sealed envelopes was used; a sealed envelope for each block contained four sequentially labelled sealed envelopes. A researcher not involved with the study and blind to the identity of the GPs undertook the randomisation. Once baseline data collection had been completed for the patients visiting a number of practices the practices were stratified according to whether they had recruited more than ten patients or less than ten patients and then allocated to a block of four. The envelope was opened and the individual practices were allocated to intervention or control depending on the contents of the four sequentially labelled envelopes. The research nurses and GPs were informed of the intervention allocation by the study administrator. The data collection staff remained blind to the allocation to prevent bias in data collection.

### Intervention

The intervention was implemented by nurses specifically recruited for this study. Nurses worked in partnership with GPs to implement the intervention. Nurses had been specifically trained to enable them to successfully implement the intervention. The training program for the nurses included attendance at a two-day workshop where the following topics were presented by expert clinicians: patho-physiology of COPD, assessment of COPD, spirometry, smoking cessation, management of COPD according to COPDX guidelines, role of pulmonary rehabilitation in the management of COPD, and the management of exacerbations.

### Intervention process

**Figure 1:**
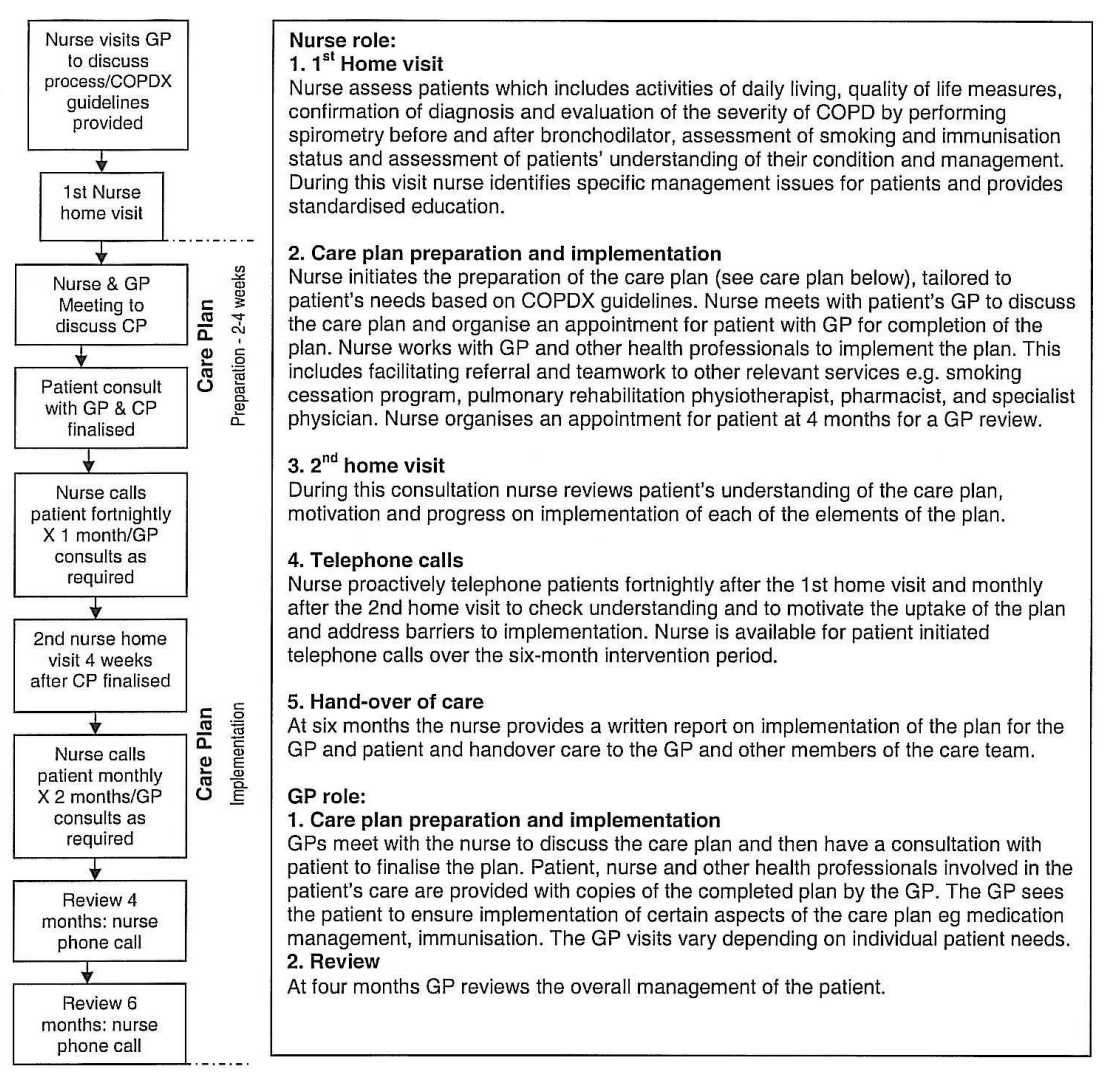


The intervention nurses worked with GPs, patients and other care providers as shown in Figure 1. Patients in the intervention group received two home visits and five telephone contacts from the nurse and a minimum of two consultations with their GP over a six-month period. The nurse and GP met face to face on two occasions and further consultation between the nurse and GP took place by telephone monthly or more frequently as needed to discuss progress and problems of the patients involved.

### Care plan content

The care plan is based on the recommendations of the COPDX evidence-based guidelines [6]. The plan aimed to optimise management, improve function, prevent deterioration and enhance patient knowledge and skills. The care plan was individualised to meet the needs of the patients and contained relevant components of the following:

1. Smoking cessation

2. Immunisation status

3. Pulmonary rehabilitation

4. Medication review

5. Nutrition

6. Psychosocial issues

7. Patient education

8. Co-morbidities and complications of COPD

#### Control Group

Patients in the control group received 'usual' care from their GPs. They were provided with a copy of the COPDX guidelines. 'Usual' care is defined as processes normally followed by the GP and the patient regarding review, pharmacological therapy and management of COPD.

#### Outcome evaluation

Quantitative outcomes were measured using instruments with demonstrated validity and reliability. A portable carbon monoxide (CO) analyser was used to validate participants' self-report of smoking status. These measures were implemented by two members of the research team who were blinded to the patients' group allocation. Data collectors received training in spirometry and use of the CO analyser.

Outcomes were measured at three points in time – at recruitment (baseline), 6 months and at 12 months after randomisation. [Table 1 shows outcomes measures being collected] These were measured at the participant's residence or at the GP practices as preferred by the patient.

**Table 1:** Schedule of data collection

	**At identification**	**At recruitment**	**6-months**	**12-months**
Age, gender, language spoken	X	X		
Demographic information		X		
Index of Comorbidity		X		
Number medication classes		X	X*	X
St George's Resp Ques		X	X	X
Spirometry		X	X	X
Immunisation status		X	X	X
Patient knowledge of COPD		X	X	X
Patient satisfaction with care		X	X	X
Smoking status: self-report		X	X	X
CO analyser		X	X	X
Health status (SF12)		X	X	X
Health service use		X	X	X
Value of partnership		X	X	X
Role and value of care plan				X

* respiratory medications only

Qualitative data about the value of the nurse-GP partnership and the role of the care plan will be collected from the intervention group by structured interviews at the conclusion of the project following collection of the 12 month quantitative outcomes.

#### Baseline measures

Patient demographic details such as age, gender, employment status, education, and country of birth are recorded at baseline. Information regarding their COPD and smoking history are also collected at baseline. Spirometry is performed to assess their COPD status and CO analyser is used to validate their smoking status. Co-morbidity is assessed using two measures: the Geriatric Index of Comorbidity [21] and the number of classes of medication used.

### Quantitative outcome measures

**Patient quality of life: (hypothesis 1) **was assessed using the St George's Respiratory Questionnaire (SGRQ) [22] at baseline and 12 month follow up. The SGRQ is a self-administered instrument designed specifically for respiratory disease. It is a 50-item instrument from which are calculated a total score and three component scores: symptoms (distress caused by respiratory symptoms), activity (physical activities thai are limited by breathlessness) and impacts (social and psychological effects of the disease). The SGRQ is scored from zero to 100 where zero indicates best quality of life and 100 worst. A change in score of four is considered to be clinically significant [22,23].

**Lung function: (hypothesis 1) **Spirometric function was measured before and 10 minutes after the administration of 200 μg sulbutamol via large volume spacer, according to standard methods. Forced expiratory volume in one second (FEV_1_) and forced vital capacity (FVC) were recorded.

***Patient health status*: (hypothesis 1) **was measured using the SF-12 [24], a generic measure of health impairment. The SF-12 gives two scores, the mental components scale and the physical component scale, which both have a mean of 50 and a standard deviation of 10.

***Health service use*: (hypothesis 1) **the number and type of general practice, specialist, community health, and hospital services used during the 12 months study period were recorded using a patient checklist of services.

***Immunisation status*: (hypothesis 2) **details of immunisation status for influenza and pneumococcal infection were collected by patient report.

***Patient knowledge *(hypothesis 2) **of the steps that can be taken to reduce the progression of COPD was assessed using the same measure as in the previous trial [20]. Patients were asked to name up to three things that they could do to prevent progression of their lung disease.

***Patient satisfaction*: (hypothesis 2) **with the overall management will be measured at 12 months using the questionnaire used in our previous study [20]. In addition, focus groups will also be conducted with a sub-set of patients in the intervention group to explore the issues even further.

***Smoking status and cessation*: (hypothesis 2) **the smoking status of patients at baseline was established by recording the number of cigarettes smoked and the length of time before the first cigarette in the morning (a measure of addiction) [25]. Attendance at a smoking cessation program and quit rates were recorded.

### Qualitative process measures

#### Value of the nurse and GP partnership

Structured interviews with the respiratory nurses and the GPs will examine satisfaction with the program and perceptions of the value of working together. Structured interviews with patients will explore the effects and value of the nurse input into COPD care. The software package NVivo^® ^will be used to facilitate coding, and exploration of the data.

#### Statistical Analysis

Data are stored in an Access database and will be exported for analysis to SPSS. Analysis will be by intention to treat. Intra-cluster (practice) correlation coefficients will be determined and published for all primary outcome variables to assist future research. Potential confounders will be compared between groups to confirm that the randomisation has provided the appropriate balance.

The effect of the intervention on outcomes measured on a continuous scale (such as SGRQ score) will be estimated and tested using mixed model analysis of variance in which time and treatment group will be fixed effects and GP practice and subject nested within practice will be random effects. The effect of the intervention on the dichotomous variables (such as smoking status, patient knowledge) will be analysed using generalised estimating equations with a logistic link and a model structure that is analogous to that described above.

#### Sample size calculations

The primary outcome variable will be 'between group' differences in post-test (12-months) is mean SGRQ scores. Data from our previous study [20] demonstrates that the between subject standard deviation in SGRQ scores is 13. The recommended minimum detectable difference to use for sample size calculations with SGRQ is 4.0 [22,23]. We have based our calculations on a intra-cluster correlation coefficient of 0.01 and a resultant design effect of 1.09 for a cluster size of 10 [26]. With this design effect the study has a > 80% power to detect a difference of 4 or greater in SGRQ. This requires at least 20 practices in each group and at least 10 patients per practice and that is at least 200 subjects in each group, (calculations in PASS software).

We estimated that the prevalence of COPD among general practice patients would be about 4.0%, slightly higher than population estimates [3]. Assuming that there are 1,500 adult patients registered with each GP then we would expect that there would be 60 patients with COPD. If a third of these patients would be eligible and consent to participate in the study, thus we will recruit 20 patients per practice. Taking a conservative approach and assuming 10 patients consent per practice, then to achieve 400 patients we will need to recruit 40 GP practices. As previously stated there are currently 785 GPs in 467 practices in SWS. Based on our previous experience of EPC evaluation [14] where 71% of the GPs contacted took part in the research we expect to be able to recruit the numbers of GPs and practices needed for this efficacy trial.

### Time plan for the study

The project was commenced in mid 2006. GP and patient recruitment were completed in February 2008. The intervention will be completed by April-May 2008 and then 6 month follow-up will commence. This will be followed by 12 month follow-up later in the year and the project will be completed by mid to late 2009.

## Competing interests

The authors declare that they have no competing interests.

## Authors' contributions

NZ – leading of development of the study conceptualisation, design, refining of protocol and write up for publication. OH – input into study conceptualisation, design and protocol publication. IH – contribution to refining and substantial contribution to writing up of the protocol for publication. EC – contribution to development of study design, advice on outcome measures and statistical issues, contribution to protocol publication. SM – input into study conceptualisation and design, expert nursing input into development of intervention, contribution to protocol publication. SV – major contribution to development of intervention, contribution to protocol publication. GM – expert respiratory medicine input into development of intervention, input into choice of outcome measures and statistical issues, contribution to protocol publication.

## Funding

The study received funding from the Australian Government National Health and Medical Research Council (NHMRC).

### Ethics Approval

The study received ethics approval from both University of New South Wales and Sydney South West Area Health Service Human Research Ethics Committees.

### Trial Registration

Australian Clinical Trials Registry (ACTR). Registration no. ACTRN012606000304538

## Pre-publication history

The pre-publication history for this paper can be accessed here:


